# Connecting the dots: linking nitrogen cycle gene expression to nitrogen fluxes in marine sediment mesocosms

**DOI:** 10.3389/fmicb.2014.00429

**Published:** 2014-08-21

**Authors:** Jennifer L. Bowen, Andrew R. Babbin, Patrick J. Kearns, Bess B. Ward

**Affiliations:** ^1^Department of Biology, University of Massachusetts BostonBoston, MA, USA; ^2^Department of Geosciences, Princeton UniversityPrinceton, NJ, USA

**Keywords:** ammonia oxidizing archaea, denitrification, nitrification, *nirS*, *amoA*, estuarine sediments, nitrogen cycle, quantitative PCR

## Abstract

Connecting molecular information directly to microbial transformation rates remains a challenge, despite the availability of molecular methods to investigate microbial biogeochemistry. By combining information on gene abundance and expression for key genes with quantitative modeling of nitrogen fluxes, we can begin to understand the scales on which genetic signals vary and how they relate to key functions. We used quantitative PCR of DNA and cDNA, along with biogeochemical modeling to assess how the abundance and expression of microbes responsible for two steps in the nitrogen cycle changed over time in estuarine sediment mesocosms. Sediments and water were collected from coastal Massachusetts and maintained in replicated 20 L mesocosms for 45 days. Concentrations of all major inorganic nitrogen species were measured daily and used to derive rates of nitrification and denitrification from a Monte Carlo-based non-negative least-squares analysis of finite difference equations. The mesocosms followed a classic regeneration sequence in which ammonium released from the decomposition of organic matter was subsequently oxidized to nitrite and then further to nitrate, some portion of which was ultimately denitrified. Normalized abundances of ammonia oxidizing archaeal ammonia monoxoygenase (*amoA*) transcripts closely tracked rates of ammonia oxidation throughout the experiment. No such relationship, however, was evident between denitrification rates and the normalized abundance of nitrite reductase (*nirS* and *nirK*) transcripts. These findings underscore the complexity of directly linking the structure of the microbial community to rates of biogeochemical processes.

## Introduction

In shallow estuarine ecosystems the biogeochemistry of sediments and the overlying water column are tightly coupled (Howarth et al., 2011). Human perturbation, however, has resulted in a substantial increase in nutrient loading to coastal waters (Bowen and Valiela, 2001; Galloway et al., 2003) resulting in a host of deleterious effects, including increases in the frequency of anoxic events (Diaz and Rosenberg, 2008) and other associated symptoms of eutrophication (Valiela et al., 1992; Cloern, 2001; Smith, 2003). Increased anthropogenic nutrient additions can alter the biogeochemical coupling between estuarine sediments and waters (Burgin and Hamilton, 2007), which can further exacerbate eutrophic conditions (Howarth et al., 2011). The microbial communities within estuarine sediments are responsible for numerous geochemical processes that can remove anthropogenic nitrogen, including canonical denitrification, coupled nitrification and denitrification, and anaerobic ammonium oxidation (anammox). As a result of these nitrogen removal pathways, microbes help to ameliorate the threat of coastal eutrophication.

The capacity of estuarine sediments to remove fixed nitrogen depends on a suite of factors. Remineralization of organic matter in estuarine sediments typically results in reducing conditions and high porewater ammonium (NH^+^_4_) concentrations. When estuarine bottom waters are oxic, some portion of NH^+^_4_ formed in the sediment is oxidized at the sediment water interface to form nitrite (NO^−^_2_) and then nitrate (NO^−^_3_) through the microbially mediated nitrification pathway. This oxidized NO^−^_3_ is then lost as nitrogen gas from the adjoining suboxic sediments through a coupling of the nitrification and denitrification pathways (Jenkins and Kemp, 1984; An and Joye, 2001; Risgaard-Petersen, 2003). In estuaries that have frequent summertime bottom water hypoxia or anoxia, the coupling of nitrification and denitrification can be interrupted as nitrification becomes inhibited by a lack of oxygen and by the accumulation of sulfide (Joye and Hollibaugh, 1995).

Canonical denitrification has typically been considered the dominant nitrogen loss process in estuarine sediments (Burdige, 2012). Rates of denitrification are controlled primarily by the absence of oxygen and the availability of both organic matter and oxidized nitrogen (Zumft, 1997). Anaerobic ammonia oxidation (anammox) can also be an important nitrogen loss process in some environments (Dalsgaard et al., 2005) but tends to account for a smaller proportion of fixed nitrogen loss in organic rich systems such as those found in the coastal zone (Dalsgaard et al., 2005; Rich et al., 2008; Nicholls and Trimmer, 2009). A third process, dissimilatory reduction of NO^−^_3_ to NH^+^_4_ (DNRA) can also occur in estuarine sediments (Giblin et al., 2013). This process does not remove nitrogen from the system, rather, it results in a change in the oxidation state of the nitrogen such that it remains bioavailable. Fixed nitrogen loss (both from anammox and denitrification) can be limited by a lack of oxidized nitrogen substrates, competition for substrate by DNRA, and sulfide inhibition (An and Gardner, 2002; Burgin and Hamilton, 2007). Although these pathways have been fairly well documented in estuarine sediments, how these important biogeochemical cycles are regulated at the microbial genetic level has received considerably less attention.

The exact nature of the coupling between geochemical rates and microbial gene expression is complex (van de Leemput et al., 2011) and appears to vary in space and time (Nogales et al., 2002; Smith et al., 2007; Abell et al., 2010; Laverock et al., 2013). Directly linking these biogeochemical processes to the genetic structure and activity of the microbial community responsible for facilitating these processes has remained a challenge. A series of reciprocal transplant experiments in estuarine sediments demonstrated that changes in microbial community composition had a direct effect on ecosystem function (Reed and Martiny, 2012), though it was not possible to directly tie these ecosystem scale effects to changes in relevant functional genes. Numerous correlational studies in coastal systems have linked functional gene abundance or expression to environmental drivers (Bernhard et al., 2007; Mosier and Francis, 2008; Abell et al., 2010), though far fewer studies include examination of these patterns over time (Laverock et al., 2013) or as a result of experimental manipulation. More quantitative data directly linking microbial genetics to geochemical fluxes are needed to improve the predictive capacity of geochemical models (Treseder et al., 2011).

Defining the relationship between microbial genetic diversity and ecosystem function is a central goal of microbial ecology (Morales and Holben, 2010). Advances in molecular methods have rapidly accelerated our understanding of microbial community structure and gene expression, yet translating shifts in microbial community structure into changes in ecosystem function remains a challenge (Knight et al., 2012; Ottesen et al., 2013). Metatranscriptomics approaches currently offer glimpses of how transcription profiles of the dominant microbial taxa respond to environmental changes (Ottesen et al., 2013) but do not allow insight into the activity of low abundance phylotypes that may also be active contributors to ecosystem function (Campbell et al., 2011; Campbell and Kirchman, 2012). Metatranscriptomic analyses are an even greater challenge in complex systems with high taxonomic richness such as those that exist in estuarine sediments.

In this study we examined the relationships among gene abundance and expression, nutrient fluxes, and modeled rates of nitrification and denitrification in a coastal sediment mesocosm experiment. The intention of this experiment was not to mimic processes as they occur in coastal sediments. Rather, it was to set in motion a chain reaction of geochemical fluxes that also occur in coastal systems, and to monitor changes in gene abundance and expression that occur coincident with changes in the mesocosm geochemistry. We hypothesized that because ammonia oxidation is largely the only metabolic option for this phylogenetically constrained group of organisms, the abundance of the ammonia monooxygenase (*amoA*) gene (and thus the ammonia oxidizing bacteria and archaea) would closely track rates of ammonia oxidation. If a tight coupling between *amoA* gene expression and ammonia oxidation rates is observed, it suggests that other ammonia loss processes (those that do not require ammonia monooxygenase or, like anammox, that occur under strictly anoxic conditions; Kartal et al., 2011), might not be important contributors to nutrient cycling in the mesocosms.

Further, we hypothesized that the correlation between the abundance of genes that encode nitrite reductase, a key enzyme in the denitrification pathway, and associated rates of denitrification would be more difficult to disentangle. Denitrifying bacteria are capable of utilizing numerous electron acceptors, including NO^−^_3_, and therefore the presence of the *nirS* gene does not necessarily indicate that active denitrification is occurring. However, we predicted that gene expression, when normalized to the total amount of the gene present in the samples, would roughly predict modeled rates, as it is only those bacteria actively expressing the genes and synthesizing proteins that are responsible for the biogeochemical process *in situ*. Establishing quantitative relationships between biogeochemical process rates of nitrification and denitrification and the underlying genetic controls on these processes could help increase the predictive power of biogeochemical models and our understanding of the marine microbial environment.

## Materials and methods

### Experimental design and sample collection

We used a benthic grab deployed from a small boat to collect surface sediment from five locations within Eel Pond in Woods Hole Massachusetts (41° 31'33 N, 70° 40'12 W) on 28 September 2008. Sediments were collected from 3 to 4 meters of water with a salinity of 28 ppt and a temperature of 19°C. None of the sediments collected exhibited signs of sulfide accumulation. Surface sediments (1–2 cm) from each grab were sectioned off with a knife, pooled together, and stored on ice until arrival at Princeton University, where they were then stored in a 12°C cold room until processing. Additionally, we collected 120 L of site water from Eel Pond, filtered it through a Whatman® GF/F filter and stored it in the dark until the mesocosms were constructed.

Four replicate mesocosms were established on 1 October 2008, each containing 3 kg of sediments overlain with 20 liters of filtered site water. Sediments from the initial grabs were thoroughly homogenized and visible macrofauna were removed. The 3 kg of sediment was then distributed evenly over the 0.1 m^2^ area of the mesocosm to a depth of approximately 2 cm. 20 L of filtered site water was then added to the mesocosm and sediments were allowed to settle for 24 h prior to sampling. Each mesocosm was fitted with a tightly sealed lid containing a two-port valve. One port of the valve was fitted with an air stone to gently circulate air through the overlying waters. The other port contained a sampling tube for removal of water for nutrient analyses. The overlying waters of the mesocosm remained oxic throughout the experiment and there was no evidence of sulfide accumulation.

The mesocosms were maintained in a darkened room for 45 days. Initial duplicate samples of sediment from the homogenized pool were collected with a 5 cc syringe corer and stored at −80°C. Two 15 mL aliquots of initial water were also collected for later nutrient analyses as described below. Every day or every other day water was withdrawn for nutrient analysis using a syringe to draw water through the sampling valve. pH and dissolved oxygen were measured on 20 mL of the withdrawn water using a YSI handheld meter. Two 15 mL aliquots were also removed and were filtered through a Whatman® GF/F filter and stored frozen for later nutrient analysis. Approximately once per week each mesocosm was opened and a sterile 5 cc syringe corer was used to remove an entire sediment column from the mesocosm. Sediment cores (3–4 cores taken through the entire depth of the sediment column) were collected, homogenized, and split between duplicate cryovials for immediate storage at −80°C.

### Nutrient analyses and modeled geochemical rates

At each time point we measured duplicate water column concentrations of NH^+^_4_, NO^−^_2_, and NO^−^_3_. Colorimetric analyses were used to measure NO^−^_2_ (Strickland and Parsons, 1972) and NH^+^_4_ (Koroleff, 1983). NO^−^_3_ concentrations were measured by chemi-luminescence after vanadium reduction (Garside, 1982; Braman and Hendrix, 1989). The measured nutrient concentration data were used to derive modeled rates of ammonification, ammonia oxidation, nitrite oxidation, and denitrification (Babbin and Ward, 2013). Briefly, a simple box model linking the three measured DIN species via these four biological processes was implemented.

To calculate rates of sediment biological N transformation from DIN measurements in the overlying water, we generated, in a Monte Carlo fashion (*n* = 5000), random sets of DIN measurements derived from the means and standard deviations of our concentration measurements. We then smoothed the concentrations with time using a Savitzky-Golay filter to minimize sampling noise. Time derivatives were numerically calculated for each of the three DIN species, and a least squares non-negative fit of rates was determined using the algorithm of Lawson and Hanson (1974) in Matlab. The Monte Carlo simulation accounted for the replicate variability in DIN concentration measurements, and the means and standard deviations of the trials are reported.

### DNA and RNA extractions

DNA was extracted in duplicate from approximately 0.5 g (wet weight) of sediment using the MoBio PowerSoil® DNA Isolation Kit (MoBio Laboratories, Carlsbad, CA) following manufacturer's instructions. Extracted DNA was purified via isopropanol precipitation and quantified using Quant-iT™ PicoGreen® dsDNA Assay (Life Technologies, Grand Island, NY). RNA was extracted from ~1 g of sediment using the MoBio RNA Powersoil® Total RNA isolation kit (MoBio Laboratories, Carlsbad CA), also following manufacturer's instructions. mRNA was quantified using a NanoDrop ND-1000 UV-Vis spectrophotmeter (Thermo Fisher Scientific, Pittsburgh, PA), and immediately reverse transcribed to cDNA using SuperScript® III First Strand Synthesis System (Invitrogen™, now Life Technologies, Grand Island, NY). Residual DNA was digested using DNase1 (New England Biolabs, Ipswich, MA) and removal of all DNA contamination was verified via PCR amplification and gel electrophoresis following manufacturer's instructions.

### Quantitative PCR

#### Denitrifier nirS qPCR

We performed qPCR of the *nirS* gene in bacterial DNA and cDNA using primers from Braker et al. (1998). The 25 μL reaction comprised 12.5 μL of SYBR®Green Brilliant III Ultra-Fast master mix (Agilent Technologies, Santa Clara, CA), 2.5 μL each of 20 μM nirS1F and nirS3R primer stocks (Braker et al., 1998), 1 μL of 1 ng/μL template DNA or cDNA, and 6.5 μL H_2_O. The qPCR reaction was carried out on a Stratagene MX-3000 (Stratagene, La Jolla, CA) with an initial denaturation step at 95°C for 15 min, followed by 40 cycles of 94°C for 15 s, 62°C for 30 s, and 72°C for 30 s. A melt curve was then performed to test the stringency of the reaction, and resulting PCR products were examined via gel electrophoresis to confirm specificity of product formation.

#### Denitrifier nirK qPCR

We performed qPCR of the *nirK* gene in bacterial DNA using the nirK1F and nirK5R primers from Braker et al. (1998). Each 25 μL reaction contained 12.5 μL SYBR®Green Brilliant III Ultra-Fast master mix (Agilent Technologies, Santa Clara, CA), 8.7 μL MilliQ water 1.25 μL each of the forward and reverse primers (0.5 μM final concentration), 0.3 μL ROX dye, and 1 μL of 10 ng μL^−1^ DNA. The qPCR reaction was performed on an Agilent MX3005p qPCR system, with an initial denaturing step at 94°C for 5 min, followed by 35 cycles of 95°C for 30 s, 58°C for 40 s and 72°C for 40 s. Melt curves were performed to test the stringency of the reaction and the PCR product size was confirmed via gel electrophoresis. Repeated attempts to quantify *nirK* in the cDNA were unsuccessful.

#### Ammonia oxidizer amoA qPCR

We performed qPCR on the *amoA* gene in DNA and cDNA from ammonia oxidizing bacteria (AOB) using previously published AOB *amoA* primers (Rotthauwe et al., 1997). Briefly, in a 20 μL reaction we added 1 μL of 12 ng/μL template DNA or cDNA, 10 μL SYBR®Green Brilliant III Ultra-Fast master mix (Agilent Technologies, Santa Clara, CA), 0.3 μL ROX dye, 1 μL each of 10 μM forward and reverse primers, 0.6 μL BSA (300 μg/mL) and 6.1 μL water. To amplify the *amoA* gene from ammonia oxidizing archaea (AOA) we also used previously published primers (Francis et al., 2005) in 20 μL reactions containing 1 μL of 12 ng/μL DNA or cDNA template, 10 μL SYBR®Green Brilliant III Ultra-Fast master mix (Agilent Technologies, Santa Clara, CA), 0.3 μL ROX, 0.2 μL each of 0.2 μM forward and reverse primers, 0.6 μL BSA (300 μg/mL), 0.5 μL MgCl_2_(3 mM final concentration) and 7.2 μL water. Both AOA and AOB qPCR reactions were performed on an Agilent MX3005p qPCR system with an initial 5 min denaturing step at 94°C, followed by 42 cycles of 94°C for 1 min, 50°C for 1.5 min, and 72°C for 1.5 min. Melt curves were again derived to test for amplification stringency, and resulting PCR products checked for specificity of product via gel electrophoresis.

Standards for all four genes (*nirS, nirK*, AOA, and AOB *amoA*) were prepared from cloned gene fragments and were serially diluted over six orders of magnitude to generate a standard curve. Gene copy numbers were calculated from Quant-iT™ PicoGreen® dsDNA Assay (Life Technologies, Grand Island, NY) quantification of the most concentrated standard. All samples from each mesocosm and for all dates for a specific gene were analyzed in triplicate on a single 96-well plate to avoid plate-to-plate variability in quantification. All plates included triplicate standard curves as well as triplicate no template controls. The Agilent software automatically generated cycle threshold values (C_T_) and, if present in the no template controls, the C_T_ values were at least five cycles higher than the C_T_ values for the lowest standard. Amplification efficiencies ranged from 80 to 94% for *nirS* and *nirK* DNA and cDNA qPCR reactions and 75–84% for AOA and AOB *amoA* qPCR reactions.

## Results

### Nutrient concentrations

**Figure 1:**
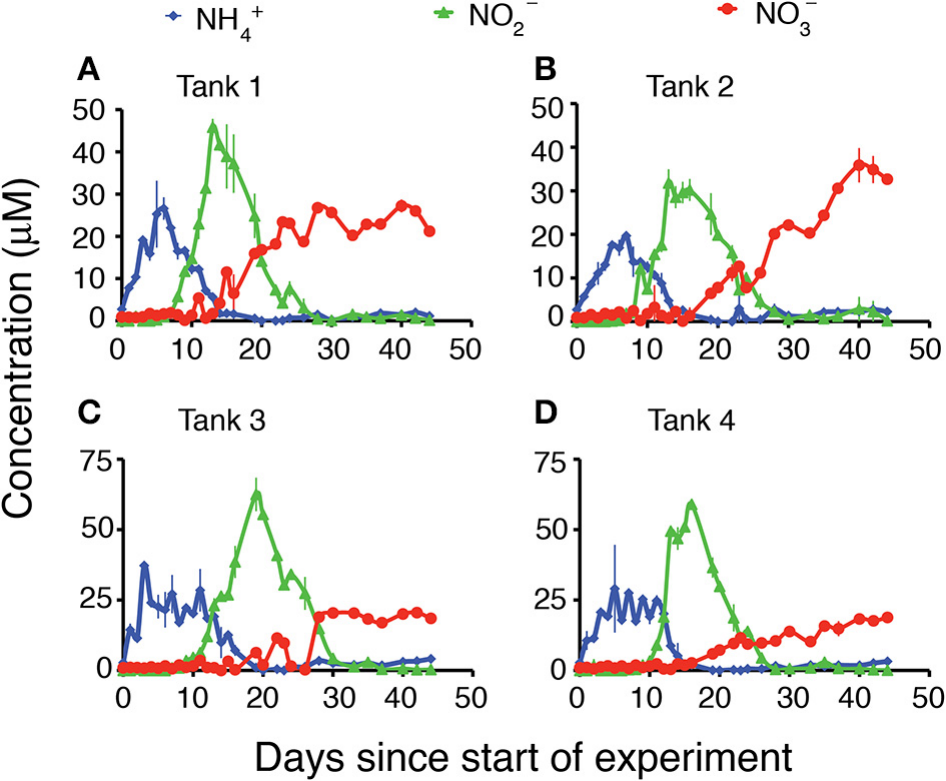
**Water column concentrations (μM) of NH_4_, NO_2_, and NO_3_ during the 45 days of the experiment**. **(A)** Tank 1, **(B)** Tank 2, **(C)** Tank 3, **(D)** Tank 4.

Each of the mesocosms followed a classic remineralization sequence, with an initial rapid flux of NH^+^_4_ resulting from decomposition of the ambient organic matter present in these rich coastal sediments at the time of sampling (Figure 1, blue diamonds). This flux of NH^+^_4_ was followed by a large flux of NO^−^_2_ (Figure 1, green triangles), and a lower and more gradual increase in NO^−^_3_ (Figure 1, red circles). The magnitude of the NH^+^_4_ concentration maximum varied from 20 to 37 μM and occurred within the first 9 days of the experiment. In all tanks NH^+^_4_ concentrations increased from approximately 3 μM in the initial water to over 10 μM within the first 2 days and remained above 10 μM for up to 2 weeks. Subsequently, typically after 9–12 days, the concentration of NO^−^_2_ began to increase to peak concentrations ranging from 31 to 62 μM. The increase in NO^−^_2_ concentration coincided with a relatively rapid decrease in NH^+^_4_. The NO^−^_2_ peaks persisted in the mesocosms until NH^+^_4_ concentrations were depleted and then NO^−^_2_ concentrations decreased to low levels (below 3 μM) for the remainder of the experiment. The concentrations of NO^−^_3_ did not show the same sharp peak as was observed with the concentrations of NH^+^_4_ or NO^−^_2_. Rather, the concentrations of NO^−^_3_ generally increased at a gradual rate throughout the duration of the experiment, with the highest concentrations of NO^−^_3_ evident at day 40 or later. All four mesocosm experiments demonstrated the same general patterns, though the timing and magnitude of peak nutrient concentrations varied slightly from tank to tank.

### Rates of nitrogen cycling

**Figure 2:**
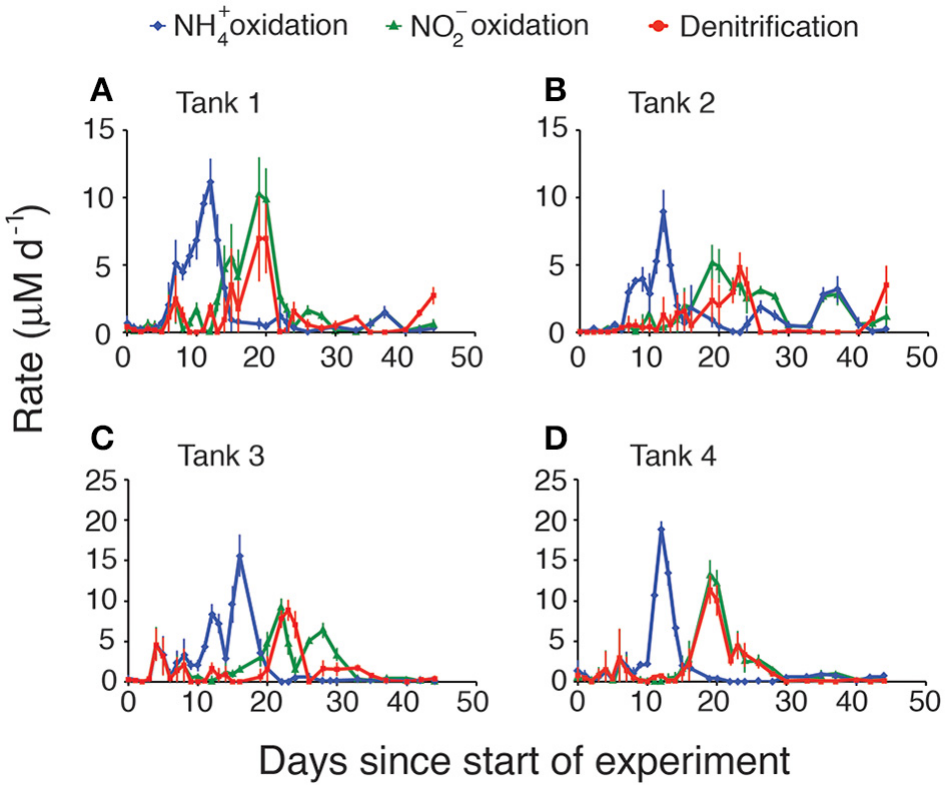
**Modeled rates of ammonia oxidation, nitrite oxidation, and denitrification in each mesocosm**. **(A)** Tank 1, **(B)** Tank 2, **(C)** Tank 3, **(D)** Tank 4.

**Figure 3:**
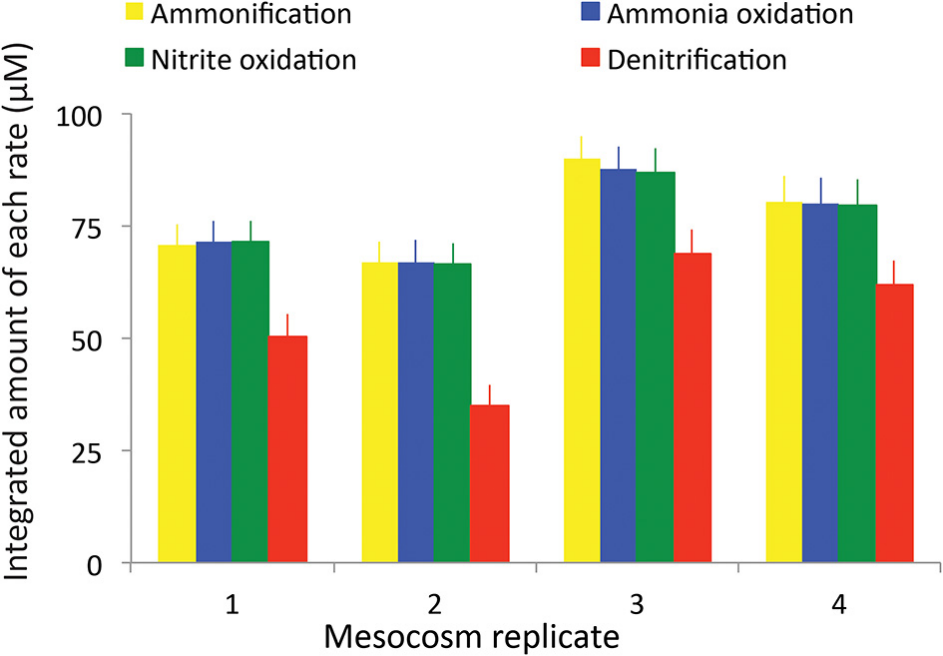
**Total integrated rates of ammonification, ammonia oxidation, nitrite oxidation, and denitrification in each mesocosm**.

Calculated rates of both stepwise components of nitrification (ammonia oxidation and nitrite oxidation) and denitrification also exhibited patterns that varied with regard to the magnitude and timing of their peaks (Figure 2), but that generally showed the same basic pattern among the four replicates. Rates were integrated over the entire sediment column and were calculated based on the changes of each nutrient in the overlying water. As expected, ammonia oxidation rates peaked first (Figure 2, blue diamonds), typically around day 10–12. The highest rates of ammonia oxidation were observed in tank 4, where they peaked at 19 μM d^−1^. In all cases there was a lag of approximately 5 days between peak ammonia oxidation rates and peak nitrite oxidation rates (Figure 2, green triangles). The magnitude of the peaks in nitrite oxidation rates was also consistently lower than the peaks in ammonia oxidation rates. Nitrite oxidation either immediately preceded or exactly co-occurred with peak rates of denitrification (Figure 2, red circles) though denitrification rates were typically slightly lower than nitrite oxidation rates. When each of the rates was integrated over time, consistent patterns emerged across all mesocosms (Figure 3). The total amount of ammonification (Figure 3, yellow bars) established the absolute amount of reactive nitrogen in the system and amounts of ammonia (Figure 3, blue bars) and nitrite oxidation (Figure 3, green bars) did not surpass the amount set by ammonification. In each case, however, the integrated amount of denitrification (Figure 3, red bars) never achieved parity with other processes. Denitrification attained only 52–76% of the maximum possible, as estimated from the amount of nitrate produced.

### Gene abundance and expression

**Figure 4:**
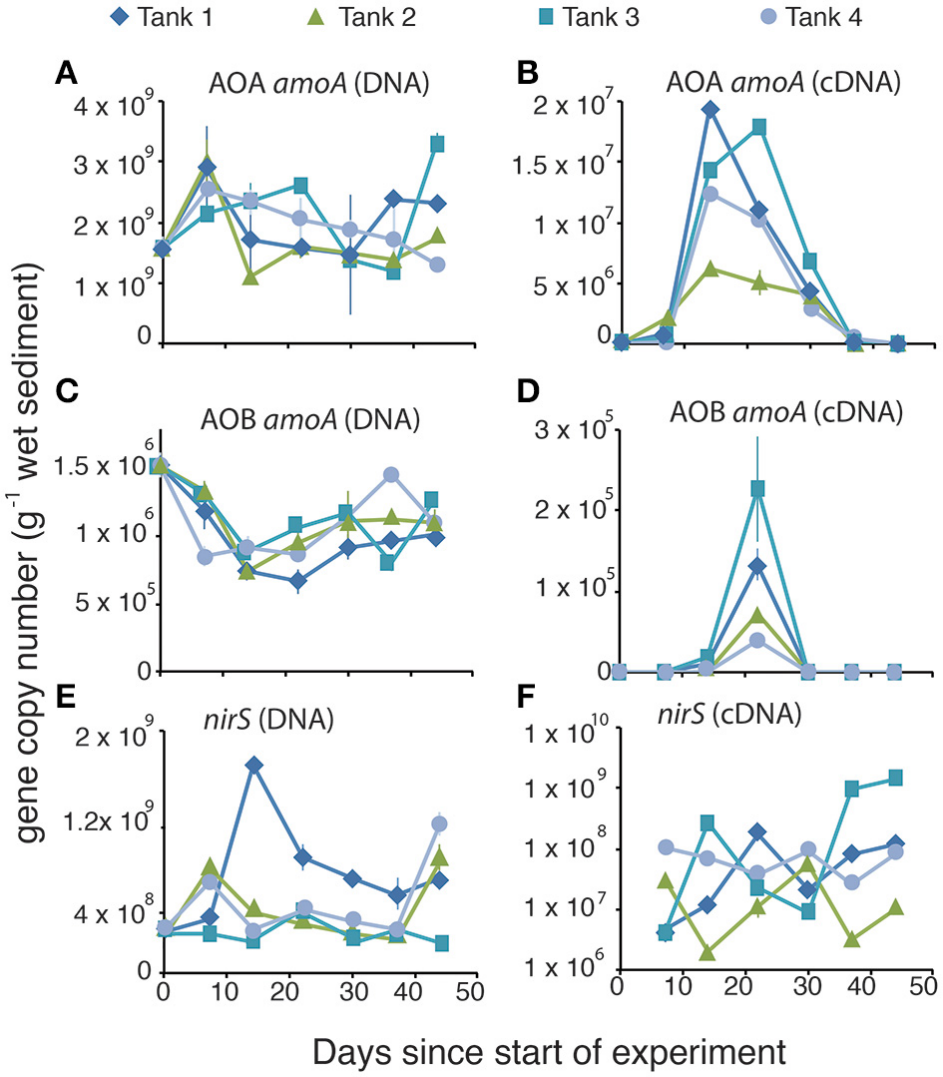
**Gene abundance (A,C,E) and gene expression (B,D,F) for ammonia oxidizing archaeal *amoA* (A,B), ammonia oxidizing bacterial *amoA* (C,D), and *nirS* denitrifiers (E,F) during the time course of the experiment**.

We used qPCR to quantify the gene abundance (via DNA) and gene expression (via cDNA) for key genes in the nitrification and denitrification pathways. In all mesocosms the abundance of AOA *amoA* in microbial DNA was around three orders of magnitude higher than the abundance of AOB *amoA* (Figure 4A vs. Figure 4C). For both AOA and AOB the abundance of *amoA* was relatively uniform throughout the experiment, displaying no temporal changes in the genetic capacity for ammonia oxidation for either domain of ammonia oxidizers (Figures 4A,C). By contrast, *amoA* gene expression for both AOA (Figure 4B) and AOB (Figure 4D) varied much more dramatically than in the DNA, with AOA peaking on day 14, and AOB peaking on day 22. Rates of AOB *amoA* gene expression were below the limits of detection, except on Days 14 and 22. Both the *nirS* (Figure 4E) and *nirK* (Figure S1) genes, which encode the two functionally redundant dissimilatory nitrite reductases in the denitrification pathway, when quantified in the DNA, were also relatively uniform. All mesocosms contained roughly similar numbers of copies of the *nirS* gene (10^8^–10^9^ copies per gram of sediment) and abundances did not change systematically through the experiment (Figure 4E). Similarly, there were no differences among the mesocosms with regard to the abundance of the *nirK* gene (Figure S1), though it was present only in ~10^5^ copies per gram of sediment, 3–4 orders of magnitude less abundant than the *nirS* gene. There were, however, widely different degrees of *nirS* gene expression (Figure 4F) among the different mesocosms with a low of 1.9 × 10^6^ copies per gram sediment in the cDNA of mesocosm #2 to a high of 1.4 × 10^9^ copies per gram of sediment in mesocosm #3. We were unable to amplify *nirK* from the cDNA, suggesting minimal expression of the *nirK* gene during this experiment.

**Figure 5:**
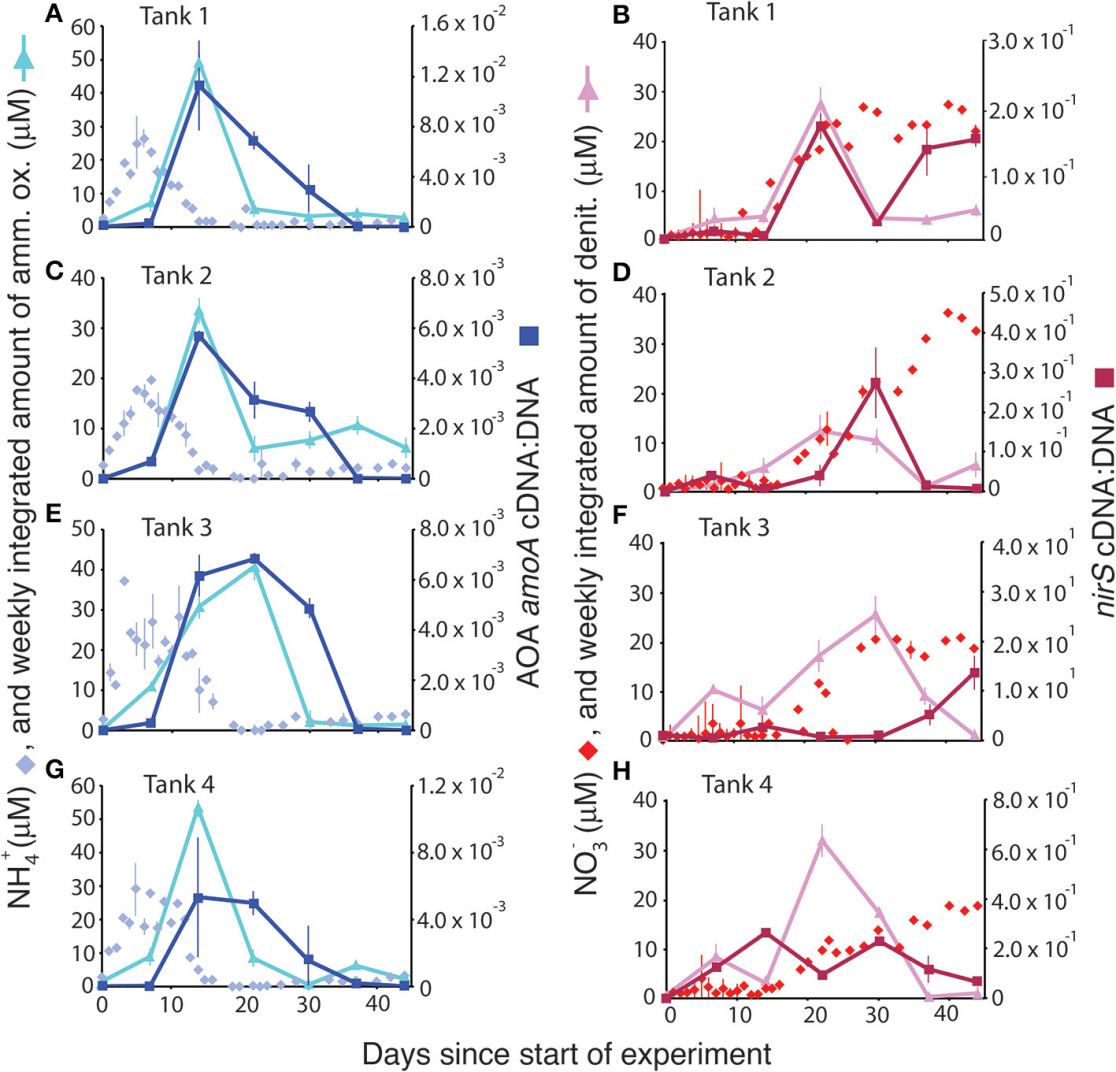
**Comparison of ammonium concentrations (μM) and ammonia oxidation rates (A,C,E,G) and nitrate concentrations (μM) and denitrification rates (B,D,F,H) with the normalized gene expression for ammonia oxidation (via AOA *amoA* cDNA:DNA) and denitrification (*nirS* cDNA:DNA)**.

Normalizing gene expression to the total gene abundance present in DNA yielded trends that closely tracked rates of ammonia oxidation (Figure 5, left panels) and that occasionally, though not typically, tracked rates of denitrification (Figure 5, right panels). In all of the mesocosms, NH^+^_4_ concentrations peaked within the first 10 days of the experiment. After a lag of approximately a week, there was a simultaneous peak in both ammonia oxidation rate and in the ratio of AOA *amoA* cDNA:DNA. NH^+^_4_ concentrations were essentially depleted by the time both rates and normalized gene expression values peaked. AOB *amoA* expression (and thus the cDNA:DNA ratio) was high only on day 22, well after the decline of the peak in ammonium concentration. The trend in normalized *nirS* gene expression occasionally mirrored denitrification rates (e.g., Figure 5A), but was considerably offset from maximal rates in most mesocosms.

**Figure 6:**
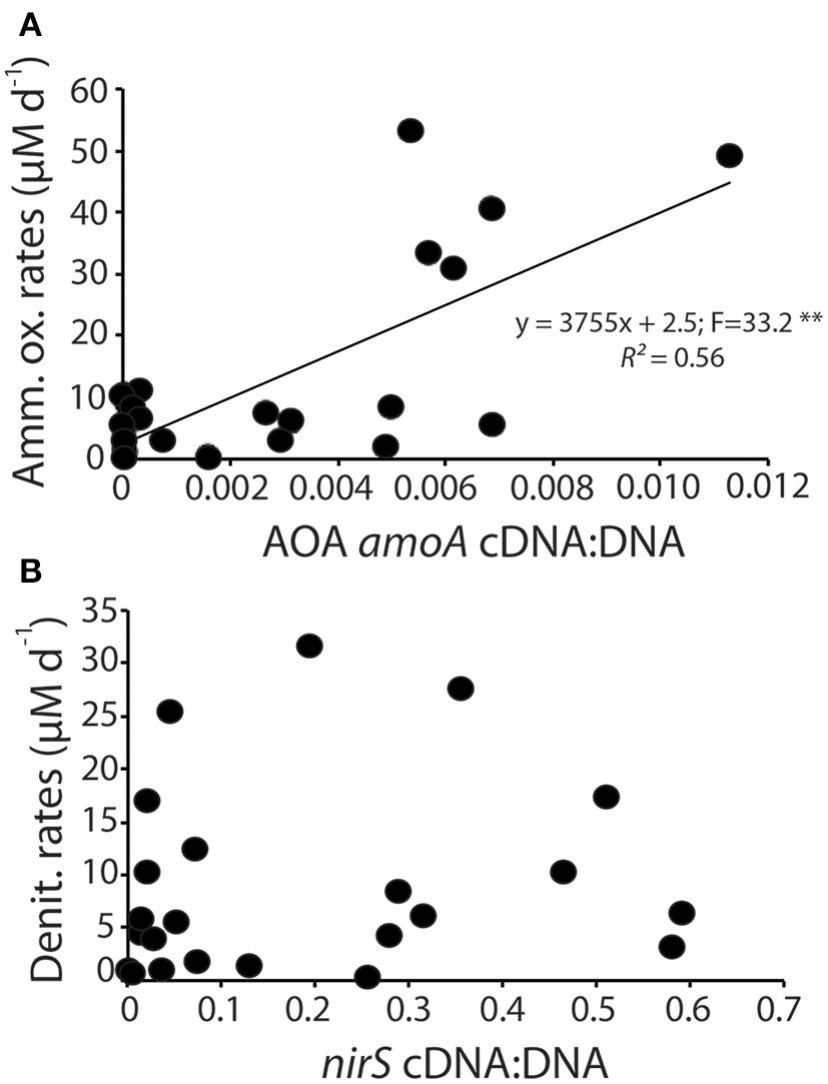
**Linear regression analysis of the normalized gene expression for AOA *amoA* compared to ammonia oxidation rates (A) and normalized *nirS* gene expression and denitrification rates (B)**. (^**^p < 0.01).

We assessed whether the normalized gene expression of ammonia oxidizers and denitrifiers, calculated as the ratio of cDNA to DNA for AOA *amoA* and *nirS*, was predictive of the modeled rates of these processes by linear regression analysis (Figure 6). Rates of ammonia oxidation did increase linearly as a function of normalized gene expression (Figure 6A) with a moderate coefficient of determination. There was no statistically significant relationship between normalized gene expression and rates of denitrification.

## Discussion

Our objective was to induce a series of biogeochemical reactions that mimic the nitrogen remineralization sequence and to determine whether changes in geochemistry would be mirrored by changes in the expression of associated genes. The ultimate goal was to determine quantifiable relationships between functional gene abundance and geochemical change to help inform geochemical models (Reed et al., 2014). The nitrogen cycle, however, is particularly complicated to model because of the numerous functional genes involved and because different taxonomic groups can dominate in different environments. For example, research in the Gulf of California showed that ammonia oxidizing archaeal (AOA) but not bacterial (AOB) gene copy number tracked ammonia oxidation rates quite closely, suggesting that the relationship between gene expression and ecosystem function is quantifiable, and dominated by AOA (Beman et al., 2008). However, in agricultural soils Jia and Conrad (2009) show that changes in ammonia oxidation rates, upon addition of NH^+^_4_, co-occurred with changes in abundance of AOB gene copy number but not AOA copy number, despite the fact that AOA were numerically much more abundant. More data on what functional genes are abundant and active under what environmental conditions are needed to better constrain geochemical models.

We hypothesized that rates of nitrification, which is assumed to be largely an obligate metabolism, would roughly track with changes in total abundance of the *amoA* gene. In general, the data confirmed this hypothesis. There was a significant linear relationship between modeled rates of ammonia oxidation and the normalized expression of the AOA *amoA* gene (Figure 6A). Interestingly, despite the largely obligate nature of the pathway, the abundance of *amoA* in the DNA of our samples, while variable, did not systematically change throughout the experiment. It is possible that some AOA in the sediments use alternative metabolisms that do not depend on *amoA* gene expression (Mußmann et al., 2011), which would further obscure the relationship between the quantity of *amoA* in DNA and the rates of ammonia oxidation. There was, however, a distinct pattern in the expression of the AOA *amoA* gene, with peaks in expression occurring about 2 weeks into the experiment. Gene expression of AOB *amoA* showed a similar sharp peak shortly after the peak in abundance of AOA *amoA* gene expression but the AOB transcripts were at least three orders of magnitude less abundant than the AOA transcripts. These results indicate that more emphasis needs to be placed on analysis of gene expression, rather than abundance, as inactive cells in the environment could obscure the linkage between gene abundance and ecosystem function.

These results highlight the importance of AOA in the nitrogen cycling of estuarine sediments, as has been previously demonstrated (Beman and Francis, 2006; Caffrey et al., 2007; Bernhard et al., 2010). Although mesocosm experiments such as these cannot be considered analogs for *in situ* processes, the results do suggest that it is primarily the AOA that are carrying out ammonia oxidation in these experiments. AOA in the initial (T0) sediments were three orders of magnitude more abundant than AOB (Figures 4A,C), indicating that the estuarine conditions at the time of collection strongly favored AOA. The numbers of AOA and AOB stayed consistent throughout the experiment, but the AOA gene expression peaked at much higher numbers and more quickly, than AOB gene expression when a source of mineralized NH^+^_4_ became available. AOB, by contrast, appear to only increase expression of their *amoA* gene after the AOA *amoA* expression decreased. It is worth noting, however, that although AOB *amoA* gene expression was much lower than AOA *amoA* gene expression, the normalized gene expression (cDNA:DNA) of AOB was approximately 0.1, compared to 0.01 for AOA, suggesting that AOB may play a disproportionately large role, relative to their total abundance, in the observed ammonia oxidation rates.

In contrast to the ammonia oxidizers, we hypothesized that the facultative denitrification pathway would be more difficult to disentangle. Since denitrifying bacteria are capable of utilizing a number of different electron acceptors, we expected that the absolute abundance of *nirS* in sediments would not track rates of denitrification, though we did expect to see a greater correlation between denitrification rates and the number or *nirS* mRNA transcripts in the sediments. In general the half-life of mRNA is relatively short (Selinger et al., 2003; Frias-Lopez et al., 2008; Steglich et al., 2010; Moran et al., 2013), and in experimental studies of the denitrifier *Pseudomonas stutzeri* the half life of *nirS* was approximately 13 min (Härtig and Zumft, 1999). The operon, however, was shown to operate nearly continuously through the 3 h experiment until resources were depleted and nitrite reduction ceased (Härtig and Zumft, 1999). Based on these experimental results we expected to see a stronger correlation between *nirS* gene expression and rates of denitrification. Our data, however, indicate that there was no consistent relationship between the normalized gene expression of the *nirS* gene and modeled rates of denitrification (Figure 6B).

Since *nirS* is one of two functionally redundant nitrite reductases encoded by prokaryotes, we hypothesized that the relationship between *nirS* and denitrification rates could be obscured by nitrogen loss by organisms containing the other nitrite reductase, *nirK*. We quantified *nirK* gene abundance in the mesocosm sediments and determined that the abundance was at least three orders of magnitude lower than the abundance of *nirS* (Figure S1). Further, we were unable to amplify *nirK* from the cDNA. We thus concluded that the abundance and activity of *nirK* denitrifiers is not likely sufficient to obscure the relationship between gene expression and modeled rates of denitrification. Nitrogen loss through the anammox reaction is another possible mechanism that could obscure the relationship between denitrification rates and *nirS* and *nirK* gene abundance. In general anammox rates are low in carbon rich environments such as are found in coastal sediments (Rich et al., 2008; Koop Jakobsen and Giblin, 2009). Furthermore, if anammox were an important process in the mesocosms it would also obscure the relationship between NH^+^_4_ oxidation rates and *amoA* gene expression because there would be an additional unaccounted for loss of NH^+^_4_. That we see a relatively tight relationship between NH^+^_4_ and *amoA* gene expression (Figure 6A), we can conclude that anammox rates are not sufficiently high to account for the lack of a relationship between *nirS* gene expression and denitrification rates. Instead we can only conclude that the facultative nature of denitrifying bacteria makes it challenging to directly link *nirS* gene expression with rates of denitrification.

Measureable accumulation of NO^−^_2_ in coastal waters is rare, although this may in part be a methodological artifact, as data are most often recorded as NO^−^_3_+NO^−^_2_. Differences in the reaction rates between ammonia oxidation and nitrite oxidation, however, can lead to transitory accumulation of NO^−^_2_. Culture experiments with *Nitrobacter*, a key nitrite oxidizer, demonstrated that high concentrations of NH^+^_4_ in the culture inhibited nitrite oxidation and resulted in the accumulation of NO^−^_2_ (Anthonisen et al., 1976). Additional modeling work indicated that the threshold concentrations for the inhibition of nitrite oxidation by free ammonia and free nitrous acid were much lower than for ammonia oxidation (Park and Bae, 2009). In our study, all four mesocosm tanks demonstrated a sharp increase in NO^−^_2_ concentrations (Figure 1), and accumulation of NO^−^_2_ in the overlying water began during times of high NH^+^_4_ concentrations. NO^−^_2_ continued to accumulate in the mesocosms until ammonia oxidizers were able to reduce available NH^+^_4_ concentrations sufficiently that inhibition of nitrite oxidizers was released.

**Figure 7:**
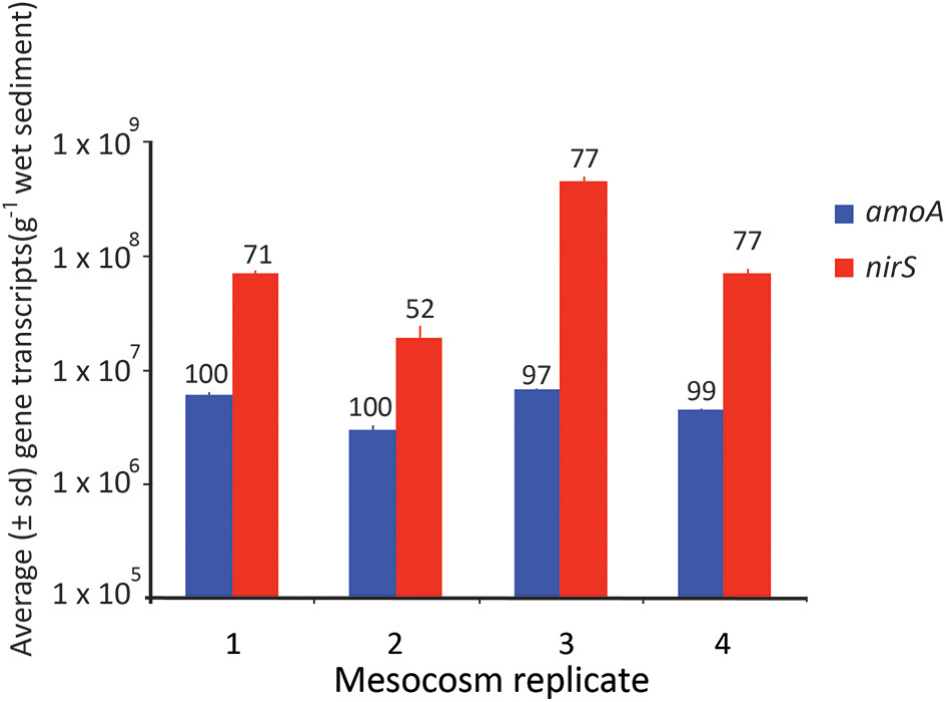
**Average (± SD) number of gene transcripts found in each mesocosm for the *amoA* and *nirS* gene**. The numbers above each bar refer to the % completion of the process facilitated by that gene.

The inhibition of nitrite oxidation by initially high NH^+^_4_ concentrations provides one plausible explanation for the overall reduced NO^−^_3_ removal capacity that was evident in the mesocosms (Figure 3). Although nearly 100% of the ammonia generated from ammonification was oxidized to NO^−^_2_ and then to NO^−^_3_, only between 52 and 76% of the oxidized NO^−^_3_ was ultimately denitrified (Figure 7) and, unlike NH^+^_4_ and NO^−^_2_, NO^−^_3_ concentrations remained high at the end of the experiment. Rates of NO^−^_3_ removal via denitrification can be limited by, among other things, the supply of NO^−^_3_ or labile organic carbon. As there was no initial NO^−^_3_ in the overlying water, the only sources of NO^−^_3_ to promote denitrification are from oxidation of accumulated NO^−^_2_ that then diffuses into the anoxic sediments, or from direct coupling of nitrification and denitrification. The accumulation of NH^+^_4_ in the overlying water and the inhibition of nitrite oxidation suggests that, initially, denitrification was limited by NO^−^_3_ supply. Further evidence for this limitation comes from the close coupling in time between rates of nitrite oxidation and rates of denitrification in the first weeks of the experiment (Figure 2).

Ultimately, however, it is likely carbon limitation that prevents complete denitrification in the mesocosms, as eventually complete oxidation of NO^−^_2_ to NO^−^_3_ did occur and NO^−^_3_ concentrations persisted in the overlying waters through the end of the experiment (Figure 1). Carbon limitation has been shown to limit denitrification in coastal sediments both in mesocosms (Babbin and Ward, 2013) and in biogeochemical models (Algar and Vallino, 2014), and is likely to be the ultimate factor limiting complete denitrification in this system. Regardless of whether it is carbon or NO^−^_3_ limitation, the limitation was ultimately observed at the genetic level. Although there was no direct relationship between denitrification rate and *nirS* gene abundance (Figure 6), when *nirS* gene expression was averaged over the time course of the experiment (Figure 7), the mean abundance of transcripts mirrored the differences in total NO^−^_3_ removal capacity, such that the mesocosm with the lowest NO^−^_3_ removal capacity also had the lowest average expression of the *nirS* gene.

Although it is difficult to extrapolate from mesocosm experiments to rates and processes in coastal sediments more broadly, these experiments bring us one step closer to understanding environmental microbial processes than experiments done solely with cultured organisms. The four mesocosms examined here replicated fairly well, with each tank demonstrating a classic regeneration sequence. The variations in the timing and magnitude of fluxes in each of the mesocosms, however, illustrate the small-scale heterogeneity that exists in coastal marine sediments. These experiments allowed us to establish a quantitative relationship between microbial community structure and ecosystem function for ammonia oxidizing archaea, but not for the much more genetically diverse denitrifiers (Bowen et al., 2013). Additional work is needed to see if the AOA/nitrification relationship is generalizable to different systems, but it is a first step in providing quantitative data on the role that these microbes play in coastal sediments and can form a basis for incorporating microbial ecology into geochemical models.

## Author contributions

Jennifer L. Bowen and Bess B. Ward designed the research, Jennifer L. Bowen collected samples, set up and monitored the mesocosms, and performed all nutrient analyses, Jennifer L. Bowen and Patrick J. Kearns did the DNA/mRNA extraction and qPCR, and analyzed the data. Andrew R. Babbin designed and implemented the model. Jennifer L. Bowen, Bess B. Ward, Andrew R. Babbin, and Patrick J. Kearns wrote the paper.

### Conflict of interest statement

The authors declare that the research was conducted in the absence of any commercial or financial relationships that could be construed as a potential conflict of interest.

## References

[B1] Abell G. C. J. Revill A. T. Smith C. Bissett A. P. Volkman J. K. Robert S. S. (2010). Archaeal ammonia oxidizers and *nirS*-type denitrifiers dominate sediment nitrifying and denitrifying populations in a subtropical macrotidal estuary. ISME J. 4, 286–300 10.1038/ismej.2009.105 19798039

[B2] Algar C. K. Vallino J. J. (2014). Predicting microbial nitrate reduction pathways in coastal sediments. Aquat. Microb. Ecol. 71, 223–238 10.3354/ame01678

[B3] An S. Gardner W. S. (2002). Dissimilatory nitrate reduction to ammonium (DNRA) as a nitrogen link, versus denitrification as a sink in a shallow estuary (Laguna Madre/Baffin Bay, Texas). Mar. Ecol. Prog. Ser. 237, 41–50 10.3354/meps237041

[B4] An S. Joye S. B. (2001). Enhancement of coupled nitrification-denitrification by benthic photosynthesis in shallow estuarine sediments. Limnol. Oceanogr. 46, 62–74 10.4319/lo.2001.46.1.0062

[B5] Anthonisen A. C. Loehr R. C. Prakasam T. B. S. Srinath E. G. (1976). inhibition of nitrification by ammonia and nitrous-acid. J. Water Pollut. Control Fed. 5, 835–852 948105

[B6] Babbin A. R. Ward B. B. (2013). Controls on nitrogen loss processes in chesapeake bay sediments. Environ. Sci. Technol. 47, 4189–4196 10.1021/es304842r 23469958

[B7] Beman J. M. Francis C. A. (2006). Diversity of ammonia-oxidizing archaea and bacteria in the sediments of a hypernutrified subtropical estuary: bahía del Tóbari, Mexico. Appl. Environ. Microbiol. 72, 7767–7777 10.1128/AEM.00946-06 17012598 PMC1694203

[B8] Beman J. M. Popp B. N. Francis C. A. (2008). Molecular and biogeochemical evidence for ammonia oxidation by marine Crenarchaeota in the Gulf of California. ISME J. 2, 429–441 10.1038/ismej.2007.118 18200070

[B9] Bernhard A. E. Landry Z. C. Blevins A. la Torre de J. R. Giblin A. E. Stahl D. A. (2010). Abundance of ammonia-oxidizing archaea and bacteria along an estuarine salinity gradient in relation to potential nitrification rates. Appl. Environ. Microbiol. 76, 1285–1289 10.1128/AEM.02018-09 20038706 PMC2820943

[B10] Bernhard A. E. Tucker J. Giblin A. E. Stahl D. A. (2007). Functionally distinct communities of ammonia-oxidizing bacteria along an estuarine salinity gradient. Environ. Microbiol. 9, 1439–1447 10.1111/j.1462-2920.2007.01260.x 17504481

[B11] Bowen J. L. Byrnes J. E. K. Weisman D. Colaneri C. (2013). Functional gene pyrosequencing and network analysis: an approach to examine the response of denitrifying bacteria to increased nitrogen supply in salt marsh sediments. Front. Microbiol. 4:342 10.3389/fmicb.2013.00342 24348464 PMC3841915

[B12] Bowen J. L. Valiela I. (2001). The ecological effects of urbanization of coastal watersheds: historical increases in nitrogen loads and eutrophication of Waquoit Bay estuaries. Can. J. Fish. Aquat. Sci. 58, 1489–1500 10.1139/f01-094

[B13] Braker G. Fesefeldt A. Witzel K.-P. (1998). Development of PCR primer systems for amplification of nitrite reductase genes (*nirK* and *nirS*) to detect denitrifying bacteria in environmental samples. Appl. Environ. Microbiol. 64, 3769–3775 9758798 10.1128/aem.64.10.3769-3775.1998 PMC106545

[B14] Braman R. S. Hendrix S. A. (1989). Nanogram nitrite and nitrate determination in environmental and biological-materials by vanadium- (III) reduction with chemi-luminescence detection. Anal. Chem. 61, 2715-2718 10.1021/ac00199a007 2619057

[B15] Burdige D. J. (2012). Estuarine and coastal sediments – coupled biogeochemical cycling, in Treatise on Estuarine and Coastal Science, eds Wolanski E. McLusky D. (Waltham, MA: Academic Press), 279–316

[B16] Burgin A. J. Hamilton S. K. (2007). Have we overemphasized the role of denitrification in aquatic ecosystems? A review of nitrate removal pathways. Front. Ecol. Environ. 5:89–96 10.1890/1540-9295(2007)5[89:HWOTRO]2.0.CO;2

[B17] Caffrey J. M. Bano N. Kalanetra K. Hollibaugh J. T. (2007). Ammonia oxidation and ammonia-oxidizing bacteria and archaea from estuaries with differing histories of hypoxia. ISME J. 1, 660–662 10.1038/ismej.2007.79 18043673

[B18] Campbell B. J. Kirchman D. L. (2012). Bacterial diversity, community structure and potential growth rates along an estuarine salinity gradient. ISME J. 7, 210–220 10.1038/ismej.2012.93 22895159 PMC3526181

[B19] Campbell B. J. Yu L. Heidelberg J. F. Kirchman D. L. (2011). Activity of abundant and rare bacteria in a coastal ocean. Proc. Natl. Acad. Sci. U.S.A. 108, 12776–12781 10.1073/pnas.1101405108 21768380 PMC3150899

[B20] Cloern J. E. (2001). Our evolving conceptual model of the coastal eutrophication problem. Mar. Ecol. Prog. Ser. 210, 223–253 10.3354/meps210223

[B21] Dalsgaard T. Thamdrup B. Canfield D. E. (2005). Anaerobic ammonium oxidation (anammox) in the marine environment. Res. Microbiol. 156, 457–464 10.1016/j.resmic.2005.01.011 15862442

[B22] Diaz R. J. Rosenberg R. (2008). Spreading dead zones and consequences for marine ecosystems. Science 321, 926–929 10.1126/science.1156401 18703733

[B23] Francis C. A. Roberts K. J. Beman J. M. Santoro A. E. Oakley B. B. (2005). Ubiquity and diversity of ammonia-oxidizing archaea in water columns and sediments of the ocean. Proc. Natl. Acad. Sci. U.S.A. 102, 14683–14688 10.1073/pnas.0506625102 16186488 PMC1253578

[B24] Frias-Lopez J. Shi Y. Tyson G. W. Coleman M. L. Schuster S. C. Chisholm S. W. (2008). Microbial community gene expression in ocean surface waters. Proc. Natl. Acad. Sci. U.S.A. 105, 3805–3810 10.1073/pnas.0708897105 18316740 PMC2268829

[B25] Galloway J. N. Aber J. D. Erisman J. W. Seitzinger S. P. Howarth R. W. Cowling E. B. (2003). The nitrogen cascade. Bioscience 53, 341–356 10.1641/0006-3568(2003)053[0341:TNC]2.0.CO;2

[B26] Garside C. (1982). A chemi-luminescent technique for the determination of nanomolar concentrations of nitrate and nitrite in seawater. Mar. Chem. 11, 159-167 10.1016/0304-4203(82)90039-1

[B27] Giblin A. E. Tobias C. R. Song B. Weston N. Banta G. T. Rivera-Monroy V. H. (2013). The importance of dissimilatory nitrate reduction to ammonium (DNRA) in the nitrogen cycle of coastal ecosystems. Oceanography 26, 124–131 10.5670/oceanog.2013.54

[B28] Härtig E. Zumft W. G. (1999). Kinetics of *nirS* expression (cytochrome *cd*_1_ nitrite reductase)in *Pseudomonas stutzeri* during the transition from aerobic respiration to denitrification: evidence for a denitrification-specific nitrate-and nitrite-responsive regulatory system. J. Bacteriol. 181, 161–166 9864326 10.1128/jb.181.1.161-166.1999 PMC103545

[B29] Howarth R. Chan F. Conley D. J. Garnier J. Doney S. C. Marino R. (2011). Coupled biogeochemical cycles: eutrophication and hypoxia in temperate estuaries and coastal marine ecosystems. Front. Ecol. Environ. 9:8 10.1890/100008

[B30] Jenkins M. C. Kemp W. M. (1984). The coupling of nitrification and denitrification in two estuarine sediments. Limnol. Oceanogr. 29, 609–619 10.4319/lo.1984.29.3.060925038845

[B31] Jia Z. Conrad R. (2009). *Bacteria* rather than *Archaea* dominate microbial ammonia oxidation in an agricultural soil. Environ. Microbiol. 11, 1658–1671 10.1111/j.1462-2920.2009.01891.x 19236445

[B32] Joye S. B. Hollibaugh J. T. (1995). Influence of sulfide inhibtion of nitrification on nitrogen regeneration in sediments. Science 270, 623–625 10.1126/science.270.5236.623

[B33] Kartal B. Maalcke W. J. de Almeida N. M. Cirpus I. Gloerich J. Geerts W. (2011). Molecular mechanism of anaerobic ammonium oxidation. Nature 479, 127–132 10.1038/nature10453 21964329

[B34] Knight R. Jansson J. Field D. Fierer N. Desai N. Fuhrman J. A. (2012). Unlocking the potential of metagenomics through replicated experimental design. Nat. Biotechnol. 30, 513–520 10.1038/nbt.2235 22678395 PMC4902277

[B35] Koop Jakobsen K. Giblin A. E. (2009). Anammox in tidal marsh sediments: the role of salinity, nitrogen loading, and marsh vegetation. Estuar. Coasts 32, 238–245 10.1007/s12237-008-9131-y

[B36] Koroleff F. (1983). Determination of nutrients, in Methods of Seawater Analysis, eds Grasshoff K. Ehrhardt M. Kremling K. (Weinheim: Verlag Chemie), 162–173

[B37] Laverock B. Tait K. Gilbert J. A. Osborn A. M. Widdicombe S. (2013). Impacts of bioturbation on temporal variation in bacterial and archaeal nitrogen-cycling gene abundance in coastal sediments. Environ. Microbiol. Rep. 6, 113–121 10.1111/1758-2229.12115 24596269 PMC4208606

[B59a] Lawson C. L. Hanson R. J. (1974). Solving Least Squares Problems. Englewood Cliffs, NJ: Prentice Hall

[B38] Morales S. E. Holben W. E. (2010). Linking bacterial identities and ecosystem processes: can “omic” analyses be more than the sum of their parts? FEMS Microbiol. Ecol. 75, 2–16 10.1111/j.1574-6941.2010.00938.x 20662931

[B39] Moran M. A. Satinksy B. Gifford S. M. Luo H. Rivers A. Chan L.-K. (2013). Sizing up metatranscriptomics. ISME J. 7, 237–243 10.1038/ismej.2012.94 22931831 PMC3554401

[B40] Mosier A. Francis C. (2008). Relative abundance and diversity of ammonia-oxidizing archaea and bacteria in the San Francisco Bay estuary. Environ. Microbiol. 10, 3002–3016 10.1111/j.1462-2920.2008.01764.x 18973621

[B41] Mußmann M. Brito I. Pitcher A. Sinninghe Damasté J. S. Hatzenpichler R. Richter A. (2011). Thaumarchaeotes abundant in refinery nitrifying sludges express *amoA* but are not obligate autotrophic ammonia oxidizers. Proc. Natl. Acad. Sci. U.S.A. 108, 16771–16776 10.1073/pnas.1106427108 21930919 PMC3189051

[B42] Nicholls J. C. Trimmer M. (2009). Widespread occurrence of the anammox reaction in estuarine sediments. Aquat. Microb. Ecol. 55, 105–113 10.3354/ame01285

[B43] Nogales B. Timmis K. N. Nedwell D. B. (2002). Detection and diversity of expressed denitrification genes in estuarine sediments after reverse transcription-PCR amplification from mRNA. Appl. Environ. Microbiol. 35, 275–298 10.1128/AEM.68.10.5017-5025.2002 12324352 PMC126436

[B44] Ottesen E. A. Young C. R. Eppley J. M. Ryan J. P. Chavez F. P. Scholin C. A. (2013). Pattern and synchrony of gene expression among sympatric marine microbial populations. Proc. Natl. Acad. Sci.U.S.A. 110, E488–E497 10.1073/pnas.1222099110 23345438 PMC3568374

[B45] Park S. Bae W. (2009). Modeling kinetics of ammonia oxidation under simultaneious inhibition by free ammonia and free nitrous acid. Process Biochem. 6, 631–640 10.1016/j.procbio.2009.02.002

[B46] Reed D. C. Algar C. K. Huber J. A. Dick G. J. (2014). Gene-centric approach to integrating environmental genomics and biogeochemical models. Proc. Natl. Acad. Sci. U.S.A. 111, 1879–1884 10.1073/pnas.1313713111 24449851 PMC3918765

[B47] Reed H. E. Martiny J. B. (2012). Microbial composition affects the functioning of estuarine sediments. ISME J. 7, 868–879 10.1038/ismej.2012.154 23235294 PMC3603390

[B48] Rich J. J. Dale O. R. Song B. Ward B. B. (2008). Anaerobic ammonium oxidation (Anammox) in Chesapeake Bay sediments. Microb. Ecol. 55, 311–320 10.1007/s00248-007-9277-3 17619213

[B49] Risgaard-Petersen N. (2003). Coupled nitrification-denitrification in autotrophic and heterotrophic estuarine sediments: on the influence of benthic microalgae. Limnol. Oceanogr. 48, 93–105 10.4319/lo.2003.48.1.0093

[B50] Rotthauwe J. H. Witzel K.-P. Liesack W. (1997). The ammonia monooxygenase structural gene *amoA* as a functional marker: molecular fine-scale analysis of natural ammonia-oxidizing populations. Appl. Environ. Microbiol. 63, 4704–4712 9406389 10.1128/aem.63.12.4704-4712.1997 PMC168793

[B51] Selinger D. W. Saxena R. M. Cheung K. J. Church G. M. Rosenow C. (2003). Global RNA half-life analysis in *Escherichia coli* reveals positional patters of transcript degradation. Genome Res. 13, 216–223 10.1101/gr.912603 12566399 PMC420366

[B52] Smith C. J. Nedwell D. B. Dong L. F. Osborne A. M. (2007). Diversity and abundance of nitrate reductase genes (*narG* and *napA*), nitrite reductase genes (*nirS* and *nrfA*), and their transcripts in estuarine sediments. Appl. Environ. Microbiol. 73, 3612–3622 10.1128/AEM.02894-06 17400770 PMC1932689

[B53] Smith V. H. (2003). Eutrophication of freshwater and coastal marine ecosystems a global problem. Environ. Sci. Pollut. Res. 10, 126–139 10.1065/espr2002.12.142 12729046

[B54] Steglich C. Lindell D. Futschik M. Rector T. Steen R. Chisholm S. W. (2010). Short RNA half-lives in the slow-growing marine cyanobacterium *Prochlorococcus*. Genome Biol. 11, R54 10.1186/gb-2010-11-5-r54 20482874 PMC2897979

[B55] Strickland J. D. H. Parsons T. R. (1972). A Practical Handbook of Seawater Analysis, 2nd Edn, Ottawa: Fisheries Research Board of Canada

[B56] Treseder K. K. Balser T. C. Bradford M. A. Brodie E. L. Dubinsky E. A. Eviner V. T. (2011). Integrating microbial ecology into ecosystem models: challenges and priorities. Biogeochemistry 109, 7–18 10.1007/s10533-011-9636-5

[B57] Valiela I. Foreman K. LaMontagne M. Hersh D. Costa J. Peckol P. (1992). Couplings of watersheds nad coastal waters -sources and consequecnes of nutrient enrichment in Waquoit Bay, Massachusetts. Estuaries 15, 443–457 10.2307/1352389

[B58] van de Leemput I. A. Veraart A. J. Dakos V. de Klein J. J. M. Strous M. Scheffer M. (2011). Predicting microbial nitrogen pathways from basic principles. Environ. Microbiol. 13, 1477–1487 10.1111/j.1462-2920.2011.02450.x 21429064

[B59] Zumft W. (1997). Cell biology and molecular basis of denitrification. Microbiol. Mol. Biol. Res. 61, 533–615 9409151 10.1128/mmbr.61.4.533-616.1997 PMC232623

