# Supplementary figures and images for: Connecting the dots: linking nitrogen cycle gene expression to nitrogen fluxes in marine sediment mesocosms
Source: Front Microbiol. 2014 Aug 21;5:429. doi: 10.3389/fmicb.2014.00429 (PMC4139956; doi:10.3389/fmicb.2014.00429)

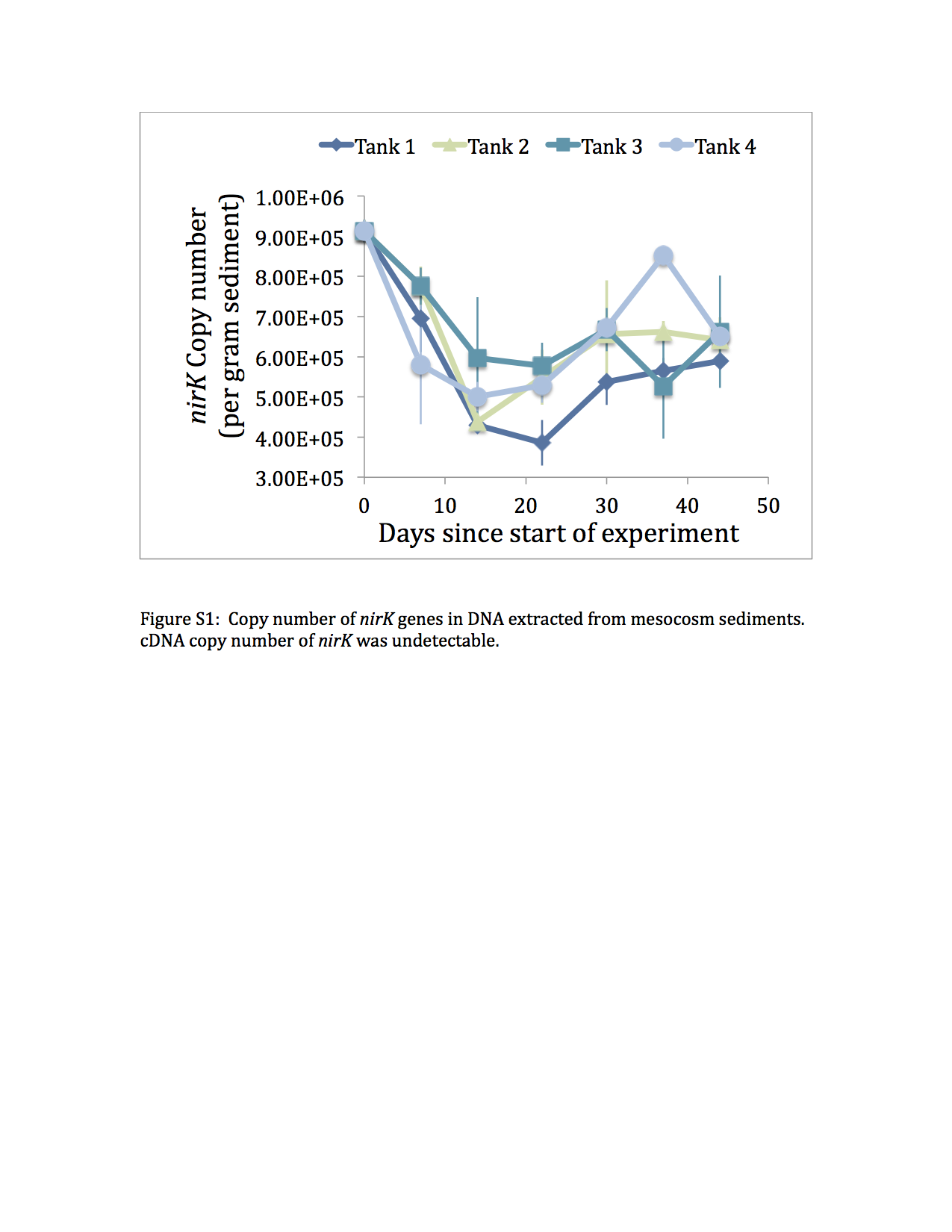

Supplement: Supplementary file 1 [file Image1.TIFF]
